# A modified method for isolation of human cardiomyocytes to model cardiac diseases

**DOI:** 10.1186/s12967-018-1649-6

**Published:** 2018-10-22

**Authors:** Guang-ran Guo, Liang Chen, Man Rao, Kai Chen, Jiang-ping Song, Sheng-shou Hu

**Affiliations:** 1 Union Hospital, Tongji Medical College, Huazhong University of Science and Technology, Wuhan, 430022 Hubei China; 2 State Key Laboratory of Cardiovascular Disease, Fuwai Hospital, National Center for Cardiovascular Diseases, Chinese Academy of Medical Sciences and Peking Union Medical College, 167A Beilishi Road, Xi Cheng District, Beijing, 100037 People’s Republic of China

**Keywords:** Human cardiomyocytes, Isolation methods, Cellular model

## Abstract

**Background:**

Cardiomyocytes derived from animals and induced pluripotent stem cells (iPSCs) are two main cellular models to study cardiovascular diseases, however, neither provides precise modeling of the response of mature human cardiomyocytes to disease or stress conditions. Therefore, there are emerging needs for finding an optimized primary human cardiomyocytes isolation method to provide a *bona fide* cellular model.

**Methods and results:**

Previous established protocols for the isolation of primary human cardiomyocytes are limited in their application due to relatively low cell yield and the requirement of tissue integrity. Here, we developed a novel, simplified method to isolate human cardiomyocytes robustly with improved viability from tissue slicing. Isolated cardiomyocytes showed intact morphology, retained contractility, ion flux, calcium handling, and responses to neurohormonal stimulation. In addition, we assessed the metabolic status of cardiomyocytes from different health conditions.

**Conclusion:**

We present a novel, simplified method for isolation of viable cardiomyocytes from human tissue.

**Electronic supplementary material:**

The online version of this article (10.1186/s12967-018-1649-6) contains supplementary material, which is available to authorized users.

## Background

A good cellular model is crucial to understanding the physiology and pathology of the heart. Due to the difficulty in obtaining heart tissue and in isolating primary cardiomyocytes, most cardiovascular studies to date adopt animal models or iPSCs-derived cardiomyocytes. However, functional and molecular characteristics of the heart vary significantly in human and animals [1]. Indeed, several studies demonstrated evidence for the marked species differences in cardiac ion currents [2, 3], and energy metabolism [4], hampering the clinical translation of findings based on such models. Although iPSCs-derived cardiomyocytes have been successfully generated from patients harboring various diseases, such cardiomyocytes are morphologically and functionally similar to fetal cells, which has become a major and common impediment to their application in modeling late-onset disorders [5]. By contrast, primary human cardiomyocytes are suitable for modeling and studying a broad spectrum of human heart diseases. Isolated myocytes with high purity avoids contamination of other cell types in heart, thereby reducing experimental noise [6].

Of note, cardiomyocytes are susceptible to hypoxia, the slightest exposure to hypoxia can cause changes in ultrastructure [7, 8], posing a great challenge to obtaining viable myocytes. Furthermore, cellular remodeling usually accompanies physiological deterioration [9], rendering myocytes particularly vulnerable to enzymatic digestion.

Enzymatic bulk digestion and the Langendorff method are two major methods for isolation of cardiac myocytes. Bulk digestion consists of enzyme digestion and mechanical agitation of the heart tissue, without requirement for specimen containing vessel structure. But owing to insufficient tissue exposure to the enzyme solution, the yield of rod-shaped myocytes was only 19.0 ± 1.6% [10]. The Langendorff method, invented in 1895, improves cell yield by using retrograde perfusion through the aorta with enzyme-containing solutions [11]. This method was initially applied to isolate cardiomyocytes from adult animal hearts and later modified to isolate human myocytes by placing a small catheter into the artery or a vein of the tissue to perfuse enzyme buffer, with reported yields of 10% to 50% rod-shaped myocytes [12]. As such, this method depends on tissue structure, and is therefore not suitable for tissue fragments from cardiac surgeries.

Considering the limitations of the two methods, we want to explore an efficient isolation method without tissue limitation. We hypothesized that the same hydrodynamic effect of perfusion could be achieved by cutting myocardium into several hundred tissue sections. Using this modified protocol, we isolated human atrial myocytes with 64.8% ± 5.0% rod-shaped cell yield.

## Methods

### Source of tissue samples

Atrial myocytes were isolated from human left atrial appendage obtained from patients undergoing coronary artery bypass grafting, ventricular tissue derived from patients with severe heart failure (HF) at the time of heart transplantation or donor hearts deemed not suitable for transplantation. We also collected the ventricular septum specimens from hypertrophic cardiomyopathy (HCM) patients. The procedure for obtaining the tissue was approved by the Ethics Committee of the Fuwai hospital and a written consent was obtained from patients.

### Transport and slice the tissue

**Fig. 1:**
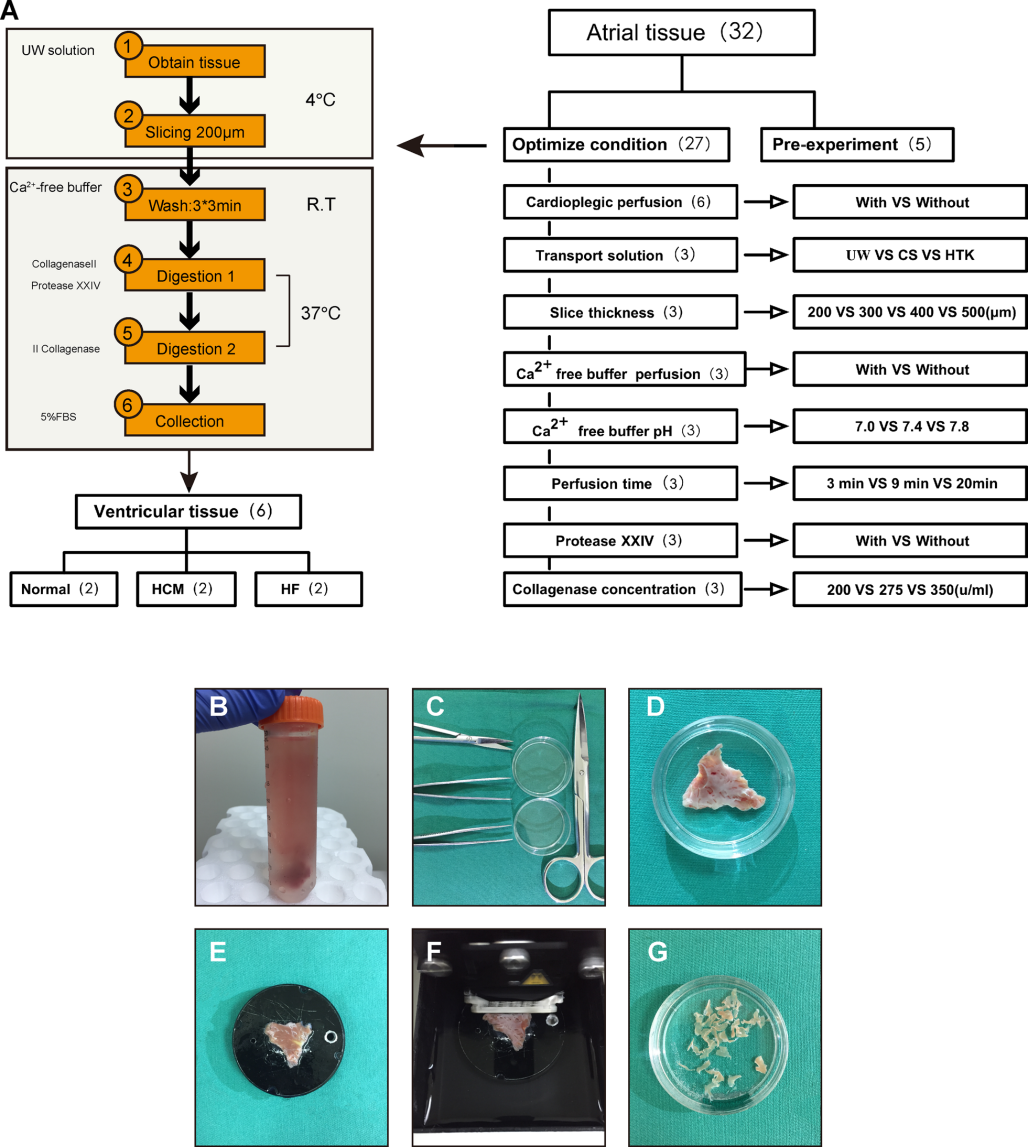
Summary of the cardiac myocyte isolation protocol. **A** A schematic overview of the cardiomyocytes isolation procedure. A more detailed description of the isolation procedure is available in Additional file 1: Experimental Procedures. **B**–**G** Photographic images of the myocardial tissue slicing procedure. **B** Transport of biopsy tissue in ice-cold UW buffer. **C** Surgical instruments for tissue dissection. **D** Tissue dissection. **e** Fixation of tissue on sectioning platform. **F** Automatic tissue slicing. **G** Resulting tissue fragments

1. Prepare 45 ml pre-chilled University of Wisconsin (UW) solution in a 50-ml conical tube and maintain it on ice for tissue transport after surgical resection.
2. Remove fat and connective tissues from the sample and clip it with sharp scissors. Smear cyanoacrylate on the epicardium side to fix tissue to the specimen plate.
3. Cut the myocardium into 200 µm-thick tissue slices using steel blades with 0.3 mm/s advance rate and vibration of 2.5 mm amplitude. The tissue should always immerse in UW buffer during sectioning. More details of this procedure can been found in Fig. 1B–G.

### Isolation procedure

#### Ca^2+^-free perfusion (9 min)

Transfer tissue fragments into a 50-ml beaker containing 15 ml of the oxygenated Ca^2+^-free solution, gently swirl at room temperature. Change to fresh buffer after 3 min and repeat twice.

#### Enzymatic digestion (0.75–1.5 h)

1. Filter the fragments and transfer them into a 50-ml conical flask containing enzymatic buffer (275 u/ml collagenase II and 1.2 u/ml protease XXIV) and swirl the flask gently.
2. Discard the supernatant once the supernatant become obscure and incubate the fragments in the same buffer.
3. Discard the supernatant and re-incubate the chunks in a fresh enzyme solution with the same composition, but without protease, which can be repeated if necessary. This step should yield large amount of rod-shaped cells.
4. Filter the cell suspension with a 100 μm filter and centrifuge the myocyte-containing conical tube at 100×*g* for 1 min to pellet myocytes. Remove the supernatant and re-suspend pelleted myocytes in 1–3 ml (depending on the yield) resuspension buffer.

### Statistical analysis

The percentage of rod-shaped cardiomyocytes is estimated by the proportion of rod-shaped myocytes in all cells, mean value is calculated from 4 fields under microscope observation. Data are presented as mean ± SEM, Statistical comparison was performed at least three independent experiments unless otherwise stated. Differences between group means were examined using two-tailed, unpaired Student’s t-test or using One Way Analysis of Variance (ANOVA) with Dunnett’s test, and were accepted as significant when P < 0.05 analyzed by SPSS software version 23.

## Results

### Isolation of cardiomyocytes from the human heart

**Fig. 2:**
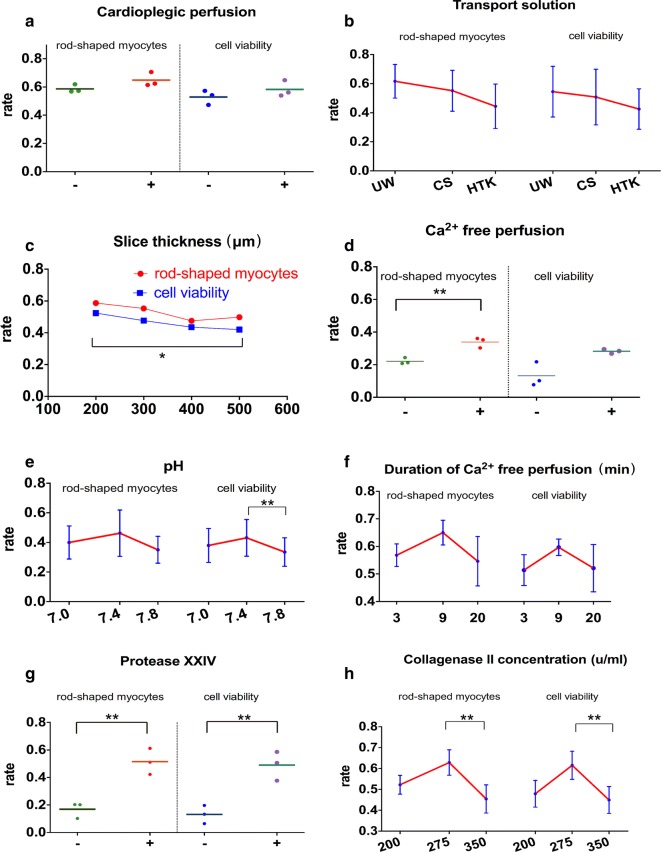
Protocol optimization. **a** Confirmation of the necessity of cardioplegic perfusion. **b** Optimization of transport solution, University of Wisconsin solution (UW), cardioplegic solution (CS), Histidine-Tryptophan-Ketoglutarate solution (HTK). **c** Optimization of slice thickness. **d** Confirmation the necessity of Ca^2+^-free buffer perfusion. **e**–**f** Optimization of Ca^2+^-free buffer pH and perfusion time. **g** Confirmation the necessity of using protease XXIV. **h** Optimization of collagenase II concentration. Quantification of the rate of rod-shaped myocytes and cell viability. Data show mean ± SD from 4 fields/condition, n = 3 independent experiments. *P < 0.05, **P < 0.01, two-tailed, unpaired Student’s *t*-test

The first 5 atrial samples were used to develop the cell isolation protocol and the rest for optimization (Fig. 1A). We found that heart tissue that underwent cardioplegic perfusion resulted in a higher yield in rod shaped and living cardiomyocytes (Fig. 2a). We also compared the effect among transport buffers UW solution, cardioplegic buffer and Histidine-Tryptophan-Ketoglutarate (HTK) solution, from which we found that the UW solution gave rise to the highest cell yield (Fig. 2b). Then we optimized slicing thickness, and demonstrated that 200 μm is superior compared to other thickness values in terms of cell yield (Fig. 2c).

We next verified the necessity of Ca^2+^ free perfusion, and observed that swirling tissue fragments in Ca^2+^ free buffer increased cell viability and shortened digestion time (Fig. 2d). Optimal rod-shaped cell yields were most reliably achieved at Ca^2+^-free buffer pH 7.4 for 9 min (3*3 min) (Fig. 2e, f).

For the choice of enzymes, we demonstrated that combining collagenase II with protease XXIV resulted in better outcome than only using collagenase (Fig. 2g), rod-shaped cardiomyocytes yield from 16.9 to 51.5% respectively. We also found the percentage of rod-shaped and viable myocytes are 62.8% and 61.5% in 275 u/ml collagenase II enzymatic buffer (Fig. 2h), higher than in 200 u/ml (52.2%, 47.9%) and 350 u/ml (45.4%, 44.9%). (The cell yield in each condition was showed in Additional file 1: Figure S1.)

### Cardiomyocytes are intact with high viability

**Fig. 3:**
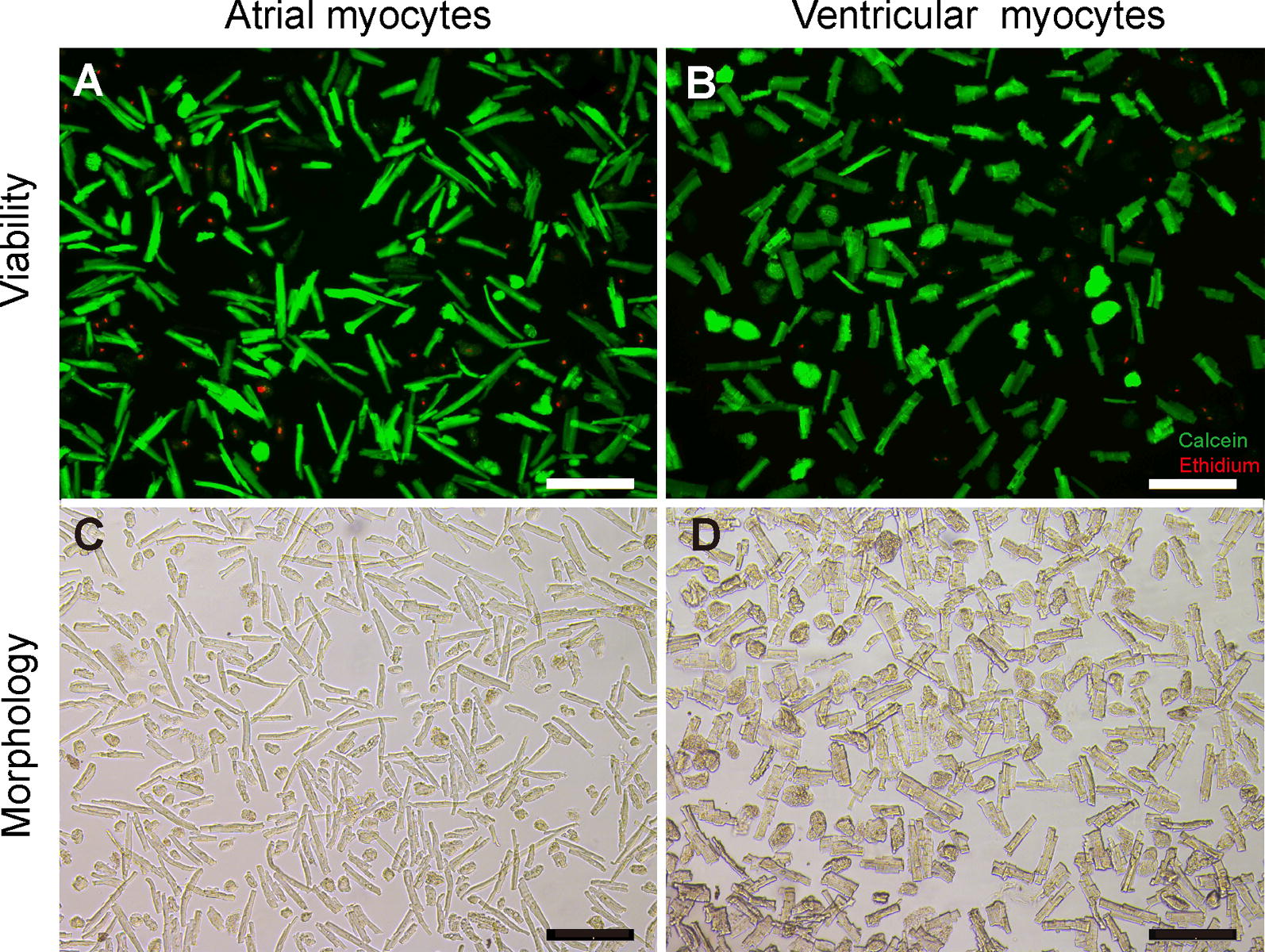
Viability (**A**, **B**) and  morphology (**C**, **D**) of cardiac myocytes. Representative images of adult atrial and ventricular digestion products from the normal heart. **A**, **B**, Cardiomyocytes immunostained for calcein (green) and ethidium (red). Images were taken with a fluorescence microscopy. Scale bars, 200 μm

The process of isolation might cause cellular damage [13]. To test the integrity of the plasma membranes of freshly isolated cardiomyocytes, a live/dead dual viability stain was used. We confirmed that most rod-shaped myocytes (64.8% ± 5.0%) were alive (58.3% ± 5.8%) with intact plasma membranes **(**Fig. 3A, C).

### Method extension from atrial tissue to disease ventricular sample

**Fig. 4:**
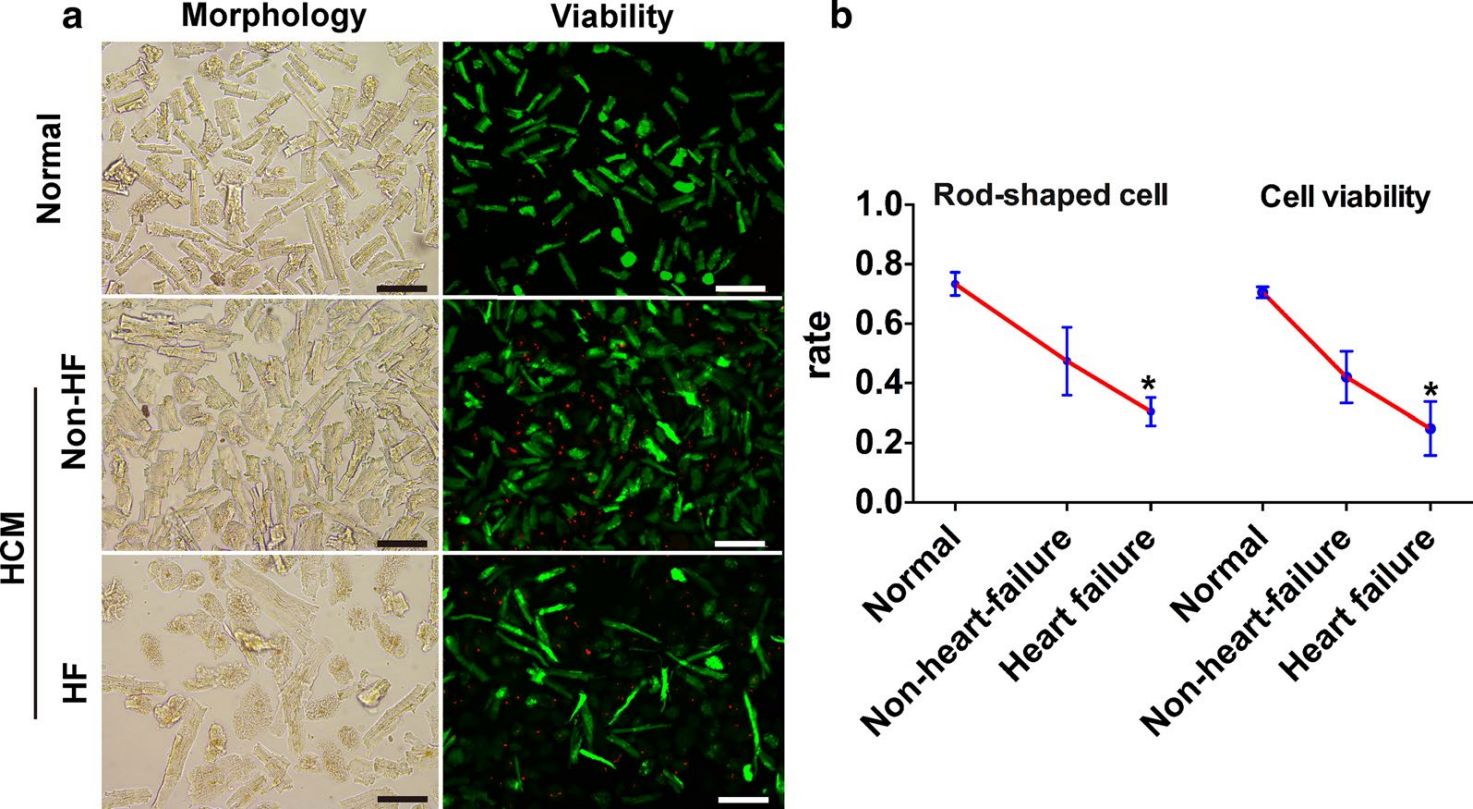
Morphology and viability of ventricular cardiomyocytes from patients with different heart diseases. **a** Representative images of ventricular myocytes from healthy controls, hypertrophic cardiomyopathy patients without or with heart failure. **b** Quantification of the rate of rod-shaped myocytes and cell viability. Data show mean ± SD, n = 2 independent experiments. Black scale bars, 100 μm, white scale bars, 200 μm, *P < 0.05 vs healthy controls

To further test whether the conditions optimized from atrial tissue are suitable for isolation of ventricular myocytes and to assess its application in diseases, we isolated ventricular myocytes from normal heart, hypertrophic cardiomyopathy (HCM) patients without heart failure (HF) and HF patients (LV ejection fraction ≤ 50%) respectively, with expected cell yields of all exceed 30% (Fig. 4a). The percentage of rod-shaped and viable myocytes are 73.4% and 70.6% from normal heart respectively, which exceeded the rates from HCM (47.4% and 42.1%) and HF (30.5% and 24.8%) patients (Fig. 4b), implying that the health status of specimen is a key factor in cell isolation efficiency.

### Cardiomyocytes retain pathologic characteristics, and are amenable to investigative techniques

**Fig. 5:**
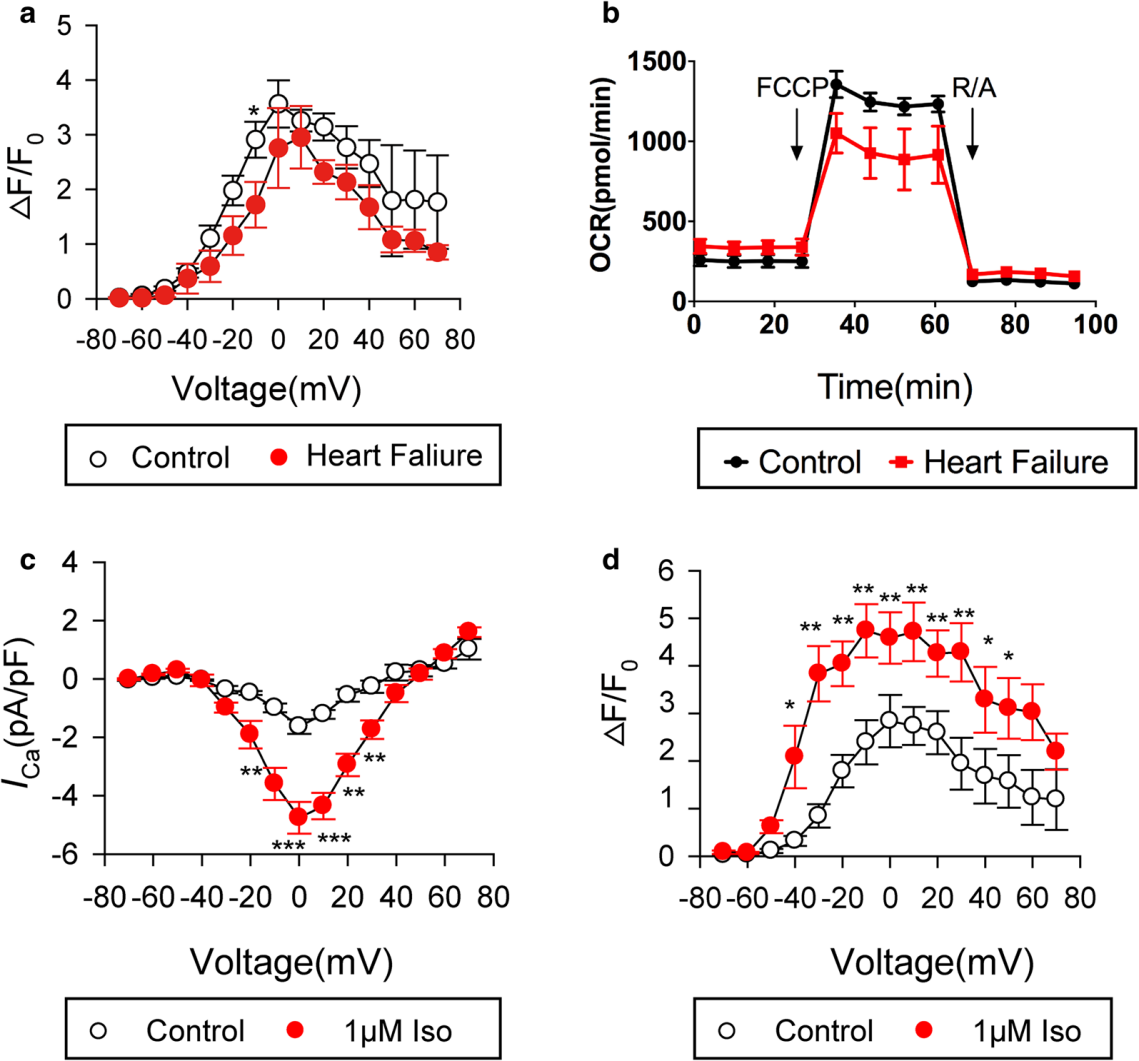
Isolated atrial cardiomyocytes retain functional characteristics and are amenable to investigation. **a** Ca^2+^ transient of cardiomyocytes from heart failure patients and non-failing controls. **b** Oxygen consumption rate (OCR) of human failing cardiomyocytes and non-failing controls. Both **c** and **d**, responsiveness of myocytes to adrenergic stimulation. **c** For whole-cell patch clamp of I_Ca,L_ recording. **d** Ca^2+^ transient recording. The Ca^2+^ concentration was reported as the fluorescence normalized to the resting level (R = F/F_0_). n = 19 cells from 2 hearts. *P < 0.05, **P < 0.01, ***P < 0.0001, two-way ANOVA multiple comparisons’ test

To determine whether isolated cardiomyocytes reflect the pathophysiological state of the heart. We isolated atrial myocytes from the HF patients and non-HF patients to compare their calcium-handling properties and metabolic statuses. With regard of Ca^2+^ transients, the reactivity of cardiomyocytes from HF patients was attenuated compared to non-HF patients (LVEF ≥ 50%) (Fig. 5a), pointing to weak contractility, consistent with published literature [14]. We also used Seahorse XF24 extracellular flux analyzer to detect the metabolic status in the two groups, and showed that failing cardiomyocytes had decreased oxygen consumption potential than non-failing myocytes (Fig. 5b), in line with the calcium-handling results.

Lastly, we evaluated the response of isolated cardiomyocytes to drug stimulation. Additional experiments were performed to confirm adrenergic responses in freshly isolated myocytes. Administration of isoproterenol amplified both calcium current and calcium transients in ventricular cardiomyocytes **(**Fig. 5c, d).

## Discussion

Enzymatic bulk digestion and the Langendorff method are two common isolation methods for heart tissue. In spite of some improvements in digestion device of bulk digestion [15], the isolation efficiency is still lower than Langendorff method. Recently, some researchers present a novel, simplified method to isolate the cardiomyocytes from adult mouse heart [13], reducing the technical difficulty. However, the requirement of tissue integrity still limits its application in human sample. Our protocol provides a optimized method to isolate cardiomyocytes from surgery waste with yields comparable to those in published Langendorff-based methods, reflecting practical advantage in processing human heart tissue.

The procedure of isolation is inevitably hypoxic; therefore, cardioprotection is the key to cell yield. We find that the more living myocytes yield from the cardioplegic perfused tissue, a step reported decreasing myocardium oxygen demand [16] as well as fatty acid oxidation [17]. For transport of myocardial specimen, most protocols opt for the Thomas solution [18] or cardioplegic buffer [19, 20] as the transport medium. It has been reported that the UW solution showed beneficial effects on the recovery of myocyte viability compared to the Thomas’ Hospital and glucose-based potassium solutions [21]. Additionally, the superior myocardial protective effects of HTK solution, as compared with conventional St. Thomas crystalloid cardioplegia has been proved [22]. We compared the effect among transport buffers HTK solution, UW solution and cardioplegic buffer, and found that the UW solution results in highest cell yield.

One step of most isolation protocols shared is a period of perfusion with a nominally Ca^2+^-free solution, in order to take advantage of the protective effect of reduced temperature, the initial washing steps, which are usually performed in Ca^2+^-free solution, was performed at room temperature, and not at 37 °C that most other protocols use [10, 23, 24]. Optimization of the entire procedure reduced isolation to around 70 min, effectively shortening ischemia time.

Human atrial tissue is readily available as atrial appendages are often discarded during surgical procedures. Therefore, initial isolation methods of adult human cardiomyocytes were established for atrial cells, and later for ventricular cells [15]. The method described here allows isolation of both atrial and ventricular myocytes with high yields as well as clear striations (Additional file 1: Figure S2). More importantly, the RNA derived from those cells is of high quality (with RNA integrity number higher than 7.5) and qualified for downstream RNA-sequencing (Additional file 1: Figure S3). A surprising finding is that some green-appearing myocytes were round, a morphology usually regarded as cells dying or being stressed or damaged [25]. We speculated these subsets of cells were still alive when exposed to the dye, but soon died and lost the rod-shape morphology. This protocol can also be applied to isolate cardiomyocytes from human heart diseases, such as heart failure, hypertrophic cardiomyopathy etc. Cell excitability (i.e. the capacity of the heart to beat spontaneously) is central to cardiac physiology [26]. We were able to record ion currents, Ca^2+^ transients and action potentials in the cells obtained via this protocol, providing a platform for patch clamp experiments [27]. In addition to excitability, cardiac energy metabolism, which mainly generates ATP from fatty acid oxidation, is also distinctly different from other organs. Alterations in myocardial energy substrate metabolism is known to occur in heart failure, switching from fatty acid oxidation toward predominantly glycolysis [28], providing opportunity for potential intervention [29]. There is compelling evidence indicating that aerobic metabolism in the mitochondria becomes increasingly important as the heart energy transitions, which can be measured via oxygen consumption rate (OCR) [30]. We successfully determined the OCR in the cardiomyocytes isolated using our method. We show that they retain the morbid metabolic characteristics, thus providing a useful tool to study heart metabolism. Finally, cells exhibited sensitivity to drug stimulation, allowing pharmacological experiments and future drug screening.

### Limitations

In fact, myocardial slices had been directly used in biochemical experiments [31]. However, similar to tissue fragments, myocardial slices still contain various cell type. Hence, data obtained from slices may be obscured by signals from cells other than myocytes.

Importantly, the success of cardiomyocytes isolation relies heavily on the speed with which we process the cells, and thus is dependent on laboratory condition, such as the distance between the operating room and the tissue culture room. Since our laboratory is convenient located near the operating area, we have the advantage to start experiment only in 3 min after biopsy. Therefore, timing requirements and expected yields need to be interpreted with precaution.

It is also noteworthy to mention that the slicing procedure might cause cell damage. Indeed, we observed many dead myocytes after the first enzymatic digestion. A cell yield was calculated before calcium re-introduction, a step resulting in 10%–15% cell death (Additional file 1: Figure S4). More work needs be done to confirm functional status of such cells during culture (Additional file 1: Figure S5).

Finally, although we demonstrated that ventricular cardiomyocytes may also be isolated using this protocol, the majority of results presented pertain to atrial myocytes, and thus need careful interpretation.

## Conclusion

The described method offers a novel approach to the isolation of viable human cardiomyocytes from surgery waste with expected cell yields. The characteristics of isolated cardiomyocytes are closer to their status in vivo and a series of functional study for cardiovascular research could be performed based on this cell model. We anticipate that this human cell model will expand and accelerate innovative research in related field.

## Additional file

**Additional file 1.** Experimental procedures and materials.   **Table S1.** Clinical characteristics of 36 patients.  **Table S2.** Troubleshooting table. **Figure S1.** Images of myocytes under conditions during optimization. **Figure S2.** Sarcomeres of cardiomyocytes. **Figure S3.** RNA integrity number of cardiomyocytes. **Figure S4.** Cell damage of calcium re-introduction. **Figure S5.**  Cardiomyocytes morphology post 48-hour culture.

## Abbreviations

iPSCs: induced pluripotent stem cells
Ca^2+^: calcium ion
UW: University of Wisconsin solution
HTK: Histidine-Tryptophan-Ketoglutarate solution
HCM: hypertrophic cardiomyopathy
HF: heart failure
ISO: isoproterenol
