# Supplementary material for: A modified method for isolation of human cardiomyocytes to model cardiac diseases
Source: J Transl Med. 2018 Oct 22;16:288. doi: 10.1186/s12967-018-1649-6 (PMC6198433; doi:10.1186/s12967-018-1649-6)
Supplement: Supplementary file 1 — Additional file 1. Experimental procedures and materials. Table S1. Clinical characteristics of 36 patients. Table S2. Troubleshooting table. Figure S1. Images of myocytes under conditions during optimization. Figure S2. Sarcomeres of cardiomyocytes. Figure S3. RNA integrity number of cardiomyocytes. Figure S4. Cell damage of calcium re-introduction. Figure S5. Cardiomyocytes morphology post 48-hour culture. [file 12967_2018_1649_MOESM1_ESM.docx]

**Additional Material**

**A Modified Method for Isolation of Human Cardiomyocytes to Model Cardiac Diseases**

**Authors:** Guang-ran Guo^1,2^, Liang Chen ^2^, Man Rao ^2^, PhD, Kai Chen ^2^, Jiang-ping Song ^2^ MD, PhD and Sheng-shou Hu ^2^ MD, PhD.

**Institution:**

1 Union Hospital, Tongji Medical College, Huazhong University of Science and Technology, Wuhan, Hubei, 430022, China.

2 State Key Laboratory of Cardiovascular Disease, Fuwai Hospital, National Center for Cardiovascular Diseases, Chinese Academy of Medical Sciences and Peking Union Medical College, 167A Beilishi Road, Xi Cheng District, Beijing 100037, China.

***Address correspondence to:**

Jiang-ping Song MD, PhD and Sheng-shou Hu MD, PhD.

State Key Laboratory of Cardiovascular Disease, Fuwai Hospital;

National Center for Cardiovascular Diseases,

Beijing, 100037, P. R. China

Tel. 0086-010-88396050, fax 086-010-88396050

Email fwsongjiangping@126.com and fwhushengshou@163.com.

**Methods**

**VibroCheck of microtome (5-7min)**

1, Insert a size 3 Allen key sideways into the blade holder through the hole and rotate it 90° clockwise.

2, Insert the provided size 3 Allen key from the top through the opening into the blade holder and open the blade holder (BH).

Critical: The clamping screw on the blade holder must not be tightened too much.

3, Hold the entire razor blade (not separated) on the left and right with both hands and insert it into the blade holder. Hook in the blade over bottom pressure plate. Clamp down the BH by turning the size 3 Allen key clockwise until hand-tight.

Critical: It is important that the blade be sharp enough and used only once, so that minimum pressure is used to make the incision.

4, Insert a size 3 Allen key sideways into the blade holder through the hole and rotate it to the desired clearance angle mark (The second line could be seen).

5, Push the VibroCheck(VC) model on the baseplate of the instrument (rear stop) and adjust the horizontal position of blade until the number shows in the screen become zero. More details can be found in Instructions for UseVT1200 S Vibrating-blade microtome

**Directly identify viability (0.6h)**

1. Dilute the calcein and ethidium dyes to final concentrations of 0.3 μmol/l and 1 μmol/l in Ca^2+^free buffer respectively.
2. Incubate the cardiomyocytes in the above buffer for 30 min at room temperature.
3. Wash the cells with Ca^2+^free buffer without dyes and re-suspend them in the same buffer without dye.
4. Calculate the percentage of green rod-shaped myocytes on a fluorescence microscope.

**Calcium re-introduction (0.75h)**

**Critical** We use 30 mM CaCl_2_ to reintroduce Ca^2+^ to a final Ca^2+^ concentration of 1.26 mM in a total volume of 5ml

i) add 14 ul of 30 mM CaCl_2_ buffer and wait for 5 min.

ii) add 28 ul of 30 mM CaCl_2_ buffer and wait for 5 min.

iii)add 56 ul of 30 mM CaCl_2_ buffer and wait for 5 min.

iv)add 112 ul of 30 mM CaCl_2_ buffer and wait for 30 min.

**Functional experiments of cardiomyocytes (**All operation was done after removing 2,3-butanedione monoxime**)**

**Patch clamp**

The patch clamp was made at room temperature using an EPC7 amplifier. Cells were bathed in extracellular solution containing (in mmol/L) 135 NaCl, 4 KCl, 1 CaCl_2_, 1 MgCl_2_, 10 Hepes, 1.2 NaH_2_PO_4_, 10 glucose, pH adjusted to 7.4 with NaOH. For measurements of the action potential (AP), current-clamp mode was employed. Cells were stimulated with current pulses 1.5 times threshold. For whole-cell patch clamp of I_Ca,L_ recording, 15 mM tetrodotoxin and 4 mM 4-aminopyridine were added in the extracellular solution. A glass pipette with a resistance (Rp) of 2-3 MΩ was filled with (in mmol/L) 110 CsCl, 6 MgCl_2_, 5 Na_2_ATP, 10 Hepes, 15 TEA•Cl, 0.2 Fluo-4 pentapotassium, pH adjusted to 7.2 with CsOH.

**Confocal Ca^2+^ imaging**

In whole-cell patch clamp experiments, the Ca^2+^ indicator Fluo-4 pentapotassium salt (10 μM) was already included in the pipette solution. Ca^2+^ imaging was recorded with a Zeiss LSM-510 inverted confocal microscope (Carl Zeiss) with 488 nm laser excitation and a 40X 1.3 N.A. oil-immersion objective.

**Measurement of oxygen consumption rates**

Oxygen consumption rates (OCR) of cardiomyocytes were measured using a Seahorse Bioscience XF24 analyzer according to Installation and Operation Manual from Seahorse Bioscience. The OCR was measured at baseline for 1h to evaluate the survival state of myocytes, and maximal OCR was measured following injection of 1 μM carbonyl cyanide 4-(trifluoromethoxy) phenylhydrazone (FCCP) dissolved in XF assay media. After 4 cycles of measuring, Rotenone (1 μM) and antimycin A (1 μM) were used to terminate mitochondrial oxygen consumption. For each experimental group, the OCR measurements were made in 4 individual wells.

**Materials**

**Reagents**

·NaCl (Sinopharm Chemical Reagent, cat. no.10019308)

·KCl（Sinopharm Chemical Reagent, cat. no.10016308）

·MgCl_2_•6H_2_O（Sinopharm Chemical Reagent, cat. no.10012828）

·NaH_2_PO_4_（Sinopharm Chemical Reagent, cat. no.20040717）

·Glucose（Sinopharm Chemical Reagent, cat. no.63005518）

·Taurine（Sinopharm Chemical Reagent, cat. no.62021434）

·Creatine（Sinopharm Chemical Reagent, cat. no.62006334）

·Pyruvate Sodium（Sinopharm Chemical Reagent, cat. no.30169134）

·KH_2_PO_4_（Sinopharm Chemical Reagent, cat. no.10017608）

·MgSO_4_ (Sinopharm Chemical Reagent, cat. no.10013016)

·Adenosine (Sinopharm Chemical Reagent, cat. no.65000531)

·Mannitol (Sinopharm Chemical Reagent, cat. no.63008816)

·CsCl (Sinopharm Chemical Reagent, cat. no. 7647178)

·CsOH (Sinopharm Chemical Reagent, cat. no. 35103798)

·NaOH (Sinopharm Chemical Reagent, cat. no. 10018560)

·KOH (Sinopharm Chemical Reagent, cat. no. 10017018)

·2,3-Butanedione Monoxime (Sigma-Aldrich, cat. no. B0753-100G)

·XXIV protease（Sigma-Aldrich, cat. no.P8038-250）

·Albumin Bovine（Roche, cat. no.10735078001）

·Collagenase II（Worthington, cat. no.LS004176）**Critical** Try to buy products with the same batch number.

·HEPES (Amresco, cat. no.7365-45-9)

· FBS (Gibco, cat. no. 10099141)

·Tetrodotoxin citrate（TOCRS, cat. no. 1069）

·4-Aminopyridine（TOCRS, cat. no.0940）

·Pentapotassium（Thermo Fisher, cat. no. F14221）

·DMEM（Thermo Fisher, cat. no. A14430-01）

·LIVE/DEAD™ Viability/Cytotoxicity Kit（Thermo Fisher ,cat. no. L3224）

·Glutamine（Thermo Fisher, cat. no. 21051024）

·XF Cell Mito Stress Test Kit（Seahorse Bioscience, cat. no. 103015-100）

**Equipment**

·Centrifuge（Eppendorf ,cat. no. 5804r ）

·50 mL centrifuge tubes（Thermo Fisher, cat. no. CLS430290）

·Ophthalmic scissors (Yuwell, cat. no. Y0010)

·Ophthalmic forceps (Yuwell, cat. no. JD1010)

·Operating scissors (Yuwell, cat. no. J21010)

·Dressing forceps (Yuwell, cat. no. J42050)

·MACS Smart Strainers (Miltenyi Biotec, cat. no. 130-098-463)

·Fluorescence microscopy (Leica, cat. no. DMI 4000B)

·Vibrating-blade microtome (Leica, cat. no. VT1200 S)

·Fluorescence microscope (Zeiss, cat. no. LSM-510)

·Seahorse XFe24 (Seahorse Bioscience, cat. no. 06415)

·XF24 Islet Capture Microplates（Seahorse Bioscience cat. no. 101122-100）

**Reagent setup**

**Calcium-free buffer** Combine the reagents listed in the table below in Milli-Q H_2_O in the order listed. Adjust the pH to 7.4 with NaOH. Store the buffer at 4 °C. The buffer is stable for up to 2 days at 4 °C.

| **Compound** | **concentration（mmol/L）** |
| --- | --- |
| NaCl | 126 |
| KCl | 4.4 |
| MgCl_2_·6H2O | 5.0 |
| NaH_2_PO_4_ | 5.0 |
| HEPES | 5.0 |
| Glucose | 22 |
| Taurine | 20 |
| Creatine | 5 |
| Pyruvate Sodium | 5 |
| 2,3-Butanedione Monoxime | 10 |

**Cardioplegic solution** Combine the reagents listed in the table below in Milli-Q H_2_O in the order listed. Adjust the pH to 7.3 with KOH. Store the solution at 4 °C. The solution is stable for up to 2 days at 4 °C.

| **Compound** | **concentration（mmol/L）** |
| --- | --- |
| KH_2_PO_4_ | 50 |
| MgSO4 | 8.0 |
| Adenosine | 5.0 |
| Mannitol | 100 |
| HEPES | 10.0 |
| Glucose | 140 |
| Taurine | 10 |

**Enzymatic buffer** Dissolve collagenase II and protease XXIV to 275 u/ml and1.2 u/ml respectively with Calcium-free buffer. The buffer is stable for 5 hours at 37 °C.

**Critical** The cell yield is expected improve with 1 mg/ml bovine serum albumin and 0.015 mmol/ml CaCl_2_ in the enzymatic buffer.

**Resuspension buffer** Calcium-free buffer contain 10% FBS. Store the buffer at 4 °C. The buffer is stable for up to 12 hours at 4 °C.

**XF assay media** XF assay media is 5.0 mM Glucose, 4.0 mM Glutamine and 5 mM Pyruvate sodium in unbuffered DMEM. Freshly prepare the solution on the day of the experiment.

**Online Tables**

**Table S1.** Clinical Characteristics of 36 Patients

| **Patient Characteristics** | |
| --- | --- |
| Age, y, mean ± SD | 60.4±7.5 |
| Male sex, n (%) | 26（72.2） |
| Cardiovascular risk factors, n (%) | |
| Hypertension | 23（63.9） |
| Diabetes | 15（41.7） |
| Hyperlipidemia | 27（75.0） |
| Medications, n (%) | |
| Aspirin | 16（44.4） |
| Statins | 34（94.4） |
| β-Blockers | 36（100.0） |
| Angiotensin-converting enzyme inhibitors | 9（25.0） |
| Calcium Channel Blockers | 30（83.3） |
| Echocardiographic data, n (%) | |
| Impaired LV function（LV ejection fraction ≤50%） | 3（8.3） |
| LV hypertrophy | 4（11.1） |
| LA hypertrophy | 9（25.0） |

**Online Table S2** Troubleshooting Table.

| **Problem** | **Possible reason** | **Solution** |
| --- | --- | --- |
| UW solution warming | The high environment temperature | Precool the trough in-20℃ |
| Poor section | Blade move too fast | Keep the speed lower than 0.5 mm/s |
|  | Fibrotic tissue | Use tweezers to pull the surface of tissue during section |
| Poor digestion | Incomplete contact with Ca^2+^ free solution | Shake the beaker to mix the cells and buffer |
|  | Old/degraded enzymes | Purchase new enzymes |
|  | New enzyme batch with low activity | Optimize enzyme concentration |
|  | Old/fibrotic heart | Increase first digestion time to 20min and enzyme concentration to 300u/ml |
| Complete digestion but low yield of rod-shaped cells | Tissue damage | Avoid mechanical damage and use the slicer correctly |
|  | Young patients | Decrease the enzyme concentration to 220u/ml |
|  | Old/contaminated buffers | Prepare new buffers |
|  | Overdigestion | Once yield a lot viable cells, stop the enzyme reaction |

**Online Figures**

**
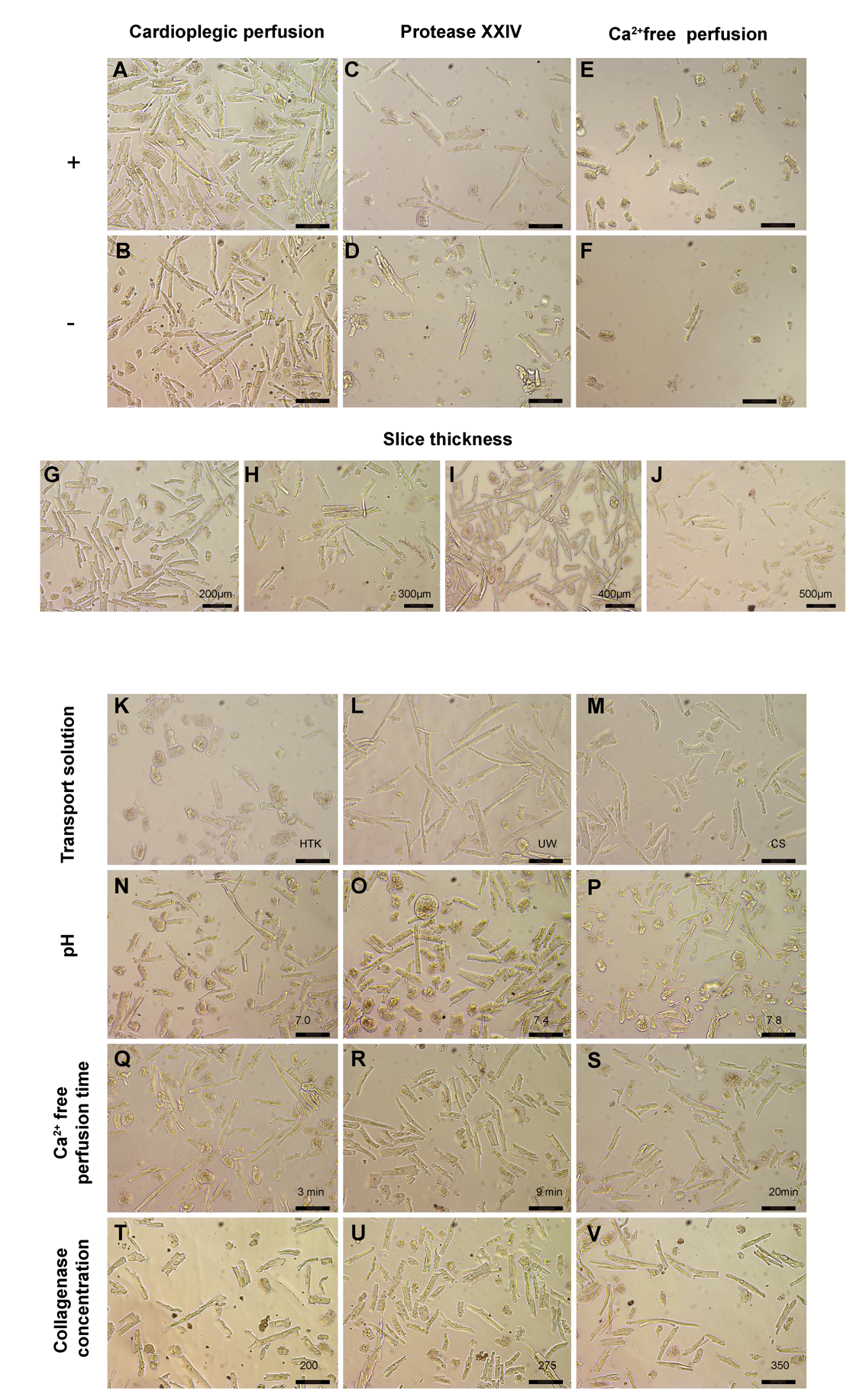
**

**Figure S1** Images of myocytes under conditions during optimization. Morphology of myocytes obtained with cardioplegic perfusion(A), protease XXIV(C) and Ca ^2+^free buffer perfusion(E) or without. From(G)to(J), Morphology of myocytes isolated from different slice thickness. Images of myocytes under different transport conditions: HTK buffer(K), UW buffer(L), Cardioplegic solution(M); pH of Ca ^2+^free buffer:7.0(N),7.4(O),7.8(P); time of Ca^2+^-free buffer perfusion: 3 min(Q), 9 min(R), 20 min(S); and collagenase II concentration: 200 u/ml(T),275 u/ml(U), 350 u/ml(V). Scale bars,100μm.

**
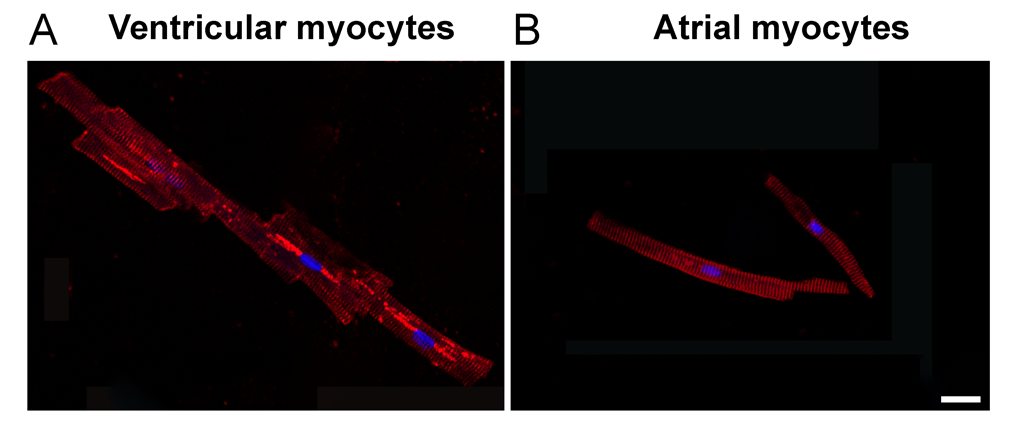
**

**Figure S2** Immunologic staining and confocal imaging of myocytes with sarcomeric-α-actinin antibody (red) and DAPI (4’,6-diamidino-2- phenylindole), Scale bars,20μm.

**Figure S3**. Bioanalyzer-based quality control of RNA integrity from cardiomyocytes isolated from heart failure patients.

**
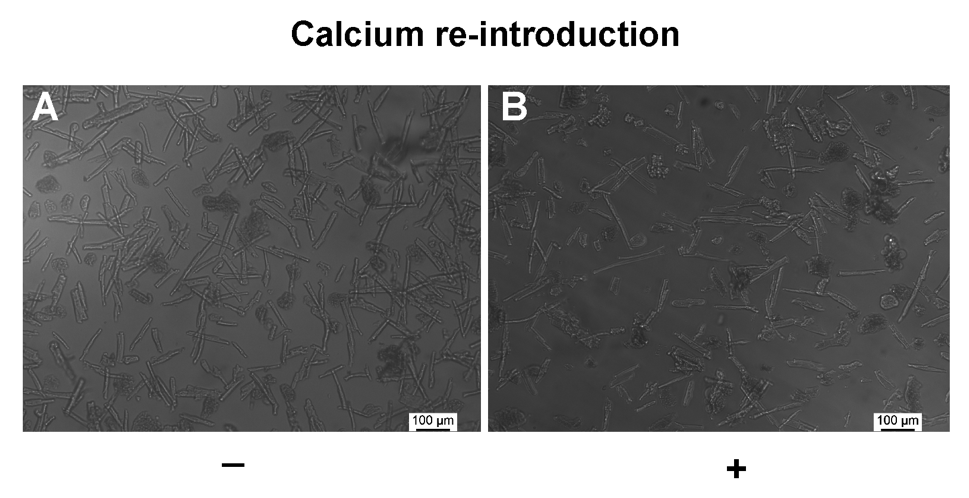
**

**Figure S4** Re-introduction of calcium causes cell damage, leading to 10%-15% cardiomyocytes death identified by trypan blue staining.


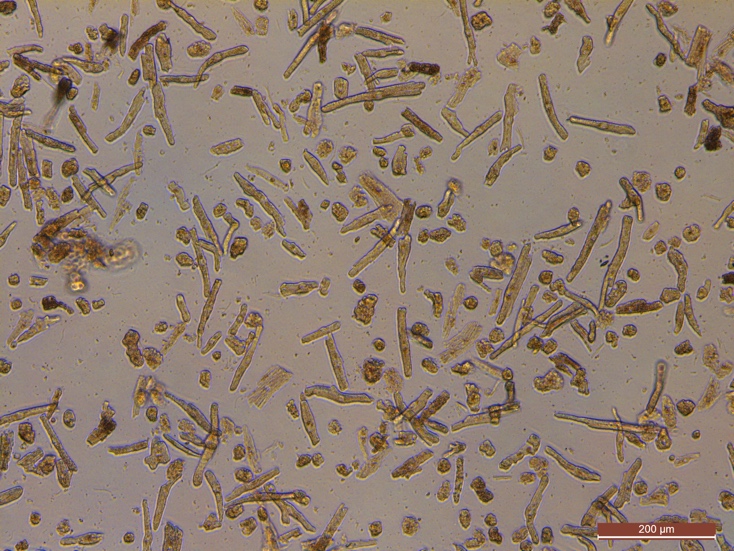


**Figure S5** The figure represented cardiomyocytes morphology post 48-hour culture.
